# Do sustainable diets take food processing into account? A scoping review

**DOI:** 10.1017/S1368980026102237

**Published:** 2026-03-03

**Authors:** Matheus Santos Cordeiro, Luana Lara Rocha, Bruna Aparecida Avelar, Alex de Oliveira Camara, Dayan Carvalho Ramos Salles de Oliveira, Mariana Carvalho de Menezes

**Affiliations:** 1 Federal University of Minas Gerais, Brazil; 2 Federal University of Ouro Preto, Brazil; 3 Fundação Oswaldo Cruz, Brazil

**Keywords:** Sustainable diets, Sustainable food systems, Food processing, Nova classification, Scoping review

## Abstract

**Objective:**

This study aimed to examine how food processing is addressed within indices/tools used to assess healthy and sustainable diets.

**Design:**

A scoping review was conducted following the PRISMA-ScR protocol. Peer-reviewed studies developing or applying indices/tools for assessing sustainable diets were included. Two independent reviewers performed the selection, with disagreements resolved by discussion, and, when necessary, a third reviewer was consulted to reach a consensus.

**Setting:**

The review included studies published in English, Portuguese or Spanish, without time restrictions and indexed in PubMed, Scopus, Web of Science and SciELO databases.

**Participants:**

A total of fifty-seven studies about sustainable diets were analysed.

**Results:**

Most studies showed significant gaps in addressing food processing and other food system components when assessing sustainable diets. The majority of studies were conducted in recent years and primarily in high-income countries, and while environmental and health dimensions of sustainability are widely explored, economic and sociocultural dimensions remain underrepresented.

**Conclusions:**

The assessment of diet sustainability remains incomplete without accounting for the role of food processing and the broader food system. There is a need for comprehensive methodologies that integrate all sustainability dimensions while also considering local contexts, particularly in low- and middle-income countries.

Sustainable food systems are essential for ensuring food security while preserving economic, social and environmental stability^(1)^. However, the global food system has been increasingly recognised as unsustainable for both the planet and its inhabitants, contributing to natural environment degradation^(2)^ through land use and deforestation, water use and greenhouse gas emissions; exacerbating climate change and biodiversity loss^(2,3)^. Additionally, food waste remains a critical issue, further undermining environmental sustainability while failing to address global nutritional needs^(4)^.

Beyond environmental impacts, current food systems present major public health challenges, particularly due to the double burden of malnutrition and climate change. The coexistence of undernutrition, including micronutrient deficiencies, and overweight within the same populations reflects structural failures in food systems that fail to ensure equitable access to healthy diets^(5,6)^. This double burden of malnutrition is directly linked to the rise of non-communicable diseases (NCD) and is further aggravated by climate change, which threatens food security by disrupting food production and increasing nutritional vulnerability, particularly in low- and middle-income countries^(5)^.

The ongoing nutrition transition, driven by urbanisation, shifts in food production systems, and the influence of the food industry has deepened dietary inequalities, particularly affecting vulnerable populations^(5,7)^. The widespread availability and consumption of ultra-processed foods (UPF) play a central role in the unsustainable trajectory of global food systems, contributing to unhealthy dietary patterns across countries^(8,9)^. UPF, which commonly contain high levels of added sugars, Na, saturated fats and artificial additives, are strongly associated with increased obesity and the prevalence of NCD^(10)^. Unequal access to healthy food is also exacerbated because UPF, being cheaper and widely available, often become the main option for low-income populations^(11)^. Beyond their health consequences, their industrial production relies on highly processed supply chains, which require monoculture farming and generate significant waste^(8,11,12)^.

This unsustainable scenario has motivated researchers worldwide to explore how current food production has led to the proliferation of low-cost, nutrient-poor foods and the increasing availability of UPF^(13)^. The NOVA food classification system, which categorises foods based on their level of processing, aligns with a food systems approach by considering both production impacts and health implications^(8,14)^. As foods undergo higher degrees of processing, they become increasingly distant from their natural state, incorporating preservatives, refined ingredients and additives that extend shelf life and enhance palatability but degrade nutritional quality^(14)^.

Evidence suggests that UPF tend to be linked to intensive resource use, and their production can contribute significantly to greenhouse gas emissions, soil degradation, biodiversity loss and packaging waste^(8,13,15,16)^. In addition, UPF supply chains are often driven by globalised commodity systems potentially increasing corporate income concentration and reducing opportunities for small-scale producers^(17)^. The growing promotion and availability of these products might also lead to the replacement of traditional diets and undermine local food cultures^(12,14)^. However, despite the increasing recognition of these impacts, the literature still lacks a comprehensive synthesis of how food processing, particularly ultra-processing, interacts with multiple dimensions of sustainability. Nevertheless, few studies have incorporated food processing as a key factor in the assessment of diet sustainability^(18,19)^.

Thus, considering the current high consumption and wide availability of UPF globally^(17,20)^, beyond the well-documented health impacts^(10,21)^, and the urgency to promote healthier and more sustainable food systems, research exploring the role of UPF across all dimensions of sustainable health diets becomes essential. This study adopts the concept proposed by FAO and WHO (2019)^(22)^, which defines sustainable healthy diets as dietary patterns that promote all dimensions of individuals’ health and well-being; have low environmental impact and are accessible, affordable, safe, equitable and culturally acceptable. These dimensions provide a comprehensive and updated framework to analyse the multiple implications of UPF consumption. Yet, food processing, as well as sociocultural and economic dimensions, remains often overlooked in studies assessing sustainable diets^(18)^.

Although some diet quality indices align indirectly with the NOVA classification^(23)^ or incorporate it directly^(24)^, they still fail to explicitly capture the multiple sustainability dimensions. Finally, existing indices for assessing sustainable diets present heterogeneous approaches and components^(25)^, reinforcing the need to map and synthesise the evaluation methods within this field. Therefore, this study aims to examine how food processing has been addressed within indices and tools used to assess healthy and sustainable diets, while also identifying how key conceptual frameworks have contributed to shaping these approaches.

## Methodology

### Literature review

The scoping review methodology was chosen due to the complexity of healthy and sustainable diets and the wide range of assessment tools proposed in the scientific literature. Additionally, with the aim of mapping the existing evidence, a broader and more detailed data extraction was sought to capture the diversity and depth of the topic. Unlike a systematic review, which aims to synthesise and evaluate the quality of evidence to answer a specific question, a scoping review seeks to map the breadth and nature of the existing literature on a given topic. This approach was therefore considered the most appropriate for identify how and to what extent food processing has been incorporated into the assessment of sustainable diets. To ensure methodological rigor, this review followed the PRISMA extension for scoping reviews (PRISMA-ScR) and the PRISMA checklist^(26)^, although no prior protocol was registered.

This approach was adopted in order to answer the following research question: ‘How is food processing considered in the concept and in the indices/tools for assessing healthy and sustainable diets?’.

Since the methodology does not involve human participants, ethical committee approval was not required.

### Bibliographic search and study selection

In this review, we adopted the broad definition of sustainable diets proposed by FAO & WHO (2019)^(22)^. Accordingly, sustainable healthy diets are understood to encompass multiple interrelated dimensions, including health and nutrition and environmental aspects; as well as sociocultural and economic aspects, such as respect for local food cultures and traditions, cultural acceptability, economic accessibility and the promotion of social equity.

The search strategy included only articles that proposed theoretical concepts and models or developed and applied indices/tools for assessing sustainable diets. Only studies published in peer-reviewed journals in Portuguese, English or Spanish were included, with no restrictions on publication date. Studies that did not assess or present concepts of sustainable diets, as well as literature reviews, commentaries and study protocols, were excluded from the review.

Given the broad and interdisciplinary nature of the topic, the search was conducted in the PubMed, SciELO, Scopus and Web of Science databases. Due to the limited availability of standardised vocabulary terms related to sustainable diets in systems such as Medical Subject Headings and Health Sciences Descriptors, various search terms were employed to capture the diverse terminology used in the literature.

The search combined terms related to healthy and sustainable diets with those associated with concepts and indices/tools for evaluating these diets. The ‘OR’ Boolean operator was used to include various indices, tools or concepts related to diet sustainability, ensuring broad coverage of different approaches. These groups of terms were then combined with the ‘AND’ operator and the main topic, ‘Sustainable Diet’, to ensure that the retrieved studies addressed both diet sustainability and the associated concepts or indices/tools (Supplementary Material 1).

The search in the databases was conducted on 20 November 2024, and the results were imported into the *Rayyan* software, where duplicates were removed. Study selection, based on titles and abstracts, was performed independently by two authors (MSC and LLR), according to the inclusion criteria. Subsequently, full texts were analysed and selected independently by the two authors. Discrepancies were resolved through discussion, and when necessary, a third author (BAA) was consulted to provide their opinion.

In line with the PRISMA-ScR extension^(26)^, a critical quality assessment of the studies was not performed. The primary objective of the scoping review was to map the available evidence, prioritising a descriptive synthesis rather than an in-depth critical analysis.

### Data collection and analysis

Data were extracted from the included articles independently by two authors (MSC and LLR), using a structured form developed in *Google Forms* to standardise the process across studies. The extracted information was subsequently compiled into a single *Excel* spreadsheet for comparison and synthesis. Discrepancies between reviewers were discussed and resolved by consensus to ensure data quality and consistency.

The extracted data included: authorship, year of publication, country/region, objective, target population, study design, methodology used, validated index/tool used, concept or recommendation of sustainable diet considered (FAO, 2012; FAO & WHO, 2019; EAT-Lancet, 2019; others), sustainable diet dimensions addressed (environmental, economic, social, cultural and health), approach to food processing (no; yes, directly; yes, indirectly), assessed food system stages (production, storage, processing, distribution, trade, preparation, consumption and waste), food groups, main findings and main limitations cited by the authors. The information extracted constituted the narrative summary of the findings.

Regarding the dimensions, a dimension was counted as ‘addressed’ when at least one indicator or aspect pertaining to that dimension was analysed. For the approach to food processing, a direct assessment was considered when the study explicitly categorised foods according to some degree of processing (e.g. the NOVA classification) or used specific metrics related to the processing stage, aiming to identify its impacts on different dimensions of diet sustainability. On the other hand, an indirect approach was considered when the study analysed impacts associated with the consumption of industrialised foods, such as discretionary foods, without classifying foods according to processing level or using specific metrics related to food processing. The food system stages were categorised based on the broad framework proposed by the HLPE (2017)^(27)^, which defines food systems as encompassing all activities from production to waste. In our analysis, we included the following stages: production (agricultural and livestock production), processing (industrial transformation), distribution (transportation), retail (price and promotion), storage (warehousing and preservation), preparation (domestic or food service-level handling), consumption (dietary intake patterns) and losses and waste (avoidable and unavoidable losses across the chain). A component was considered ‘addressed’ when the study included at least one indicator, outcome or discussion related to that stage.

## Results

**Figure 1.**
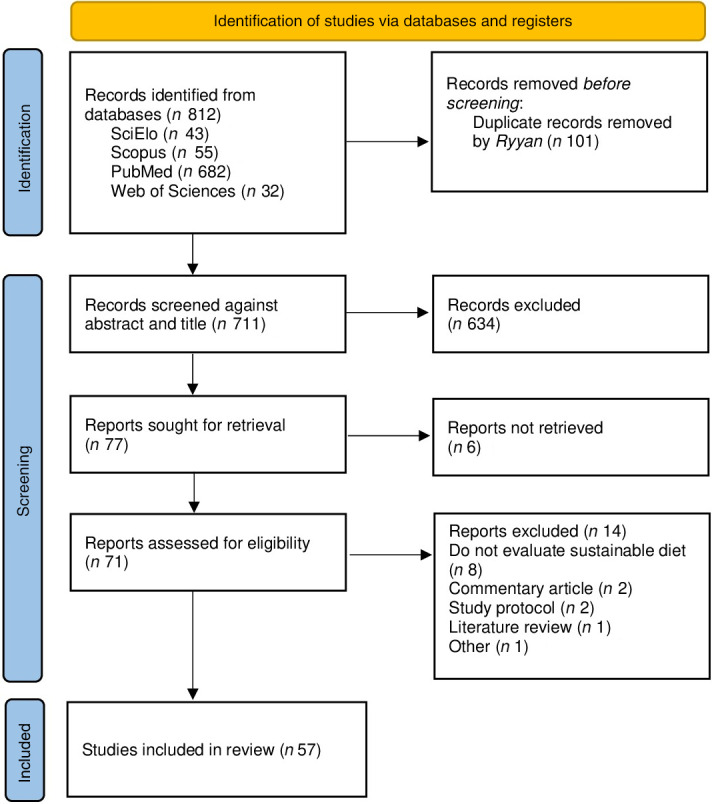
PRISMA-ScR flow diagram of the selection process.

After searching the databases, 812 studies were retrieved, of which 101 duplicates were removed. The initial screening, based on titles and abstracts, excluded 634 studies, leaving seventy-seven for full-text review. At the end of the process, fifty-seven studies met the inclusion criteria and were incorporated into the review (Figure 1). A detailed list of included articles, along with descriptions of their main characteristics, can be found in the Supplementary Material.

**Figure 2.**
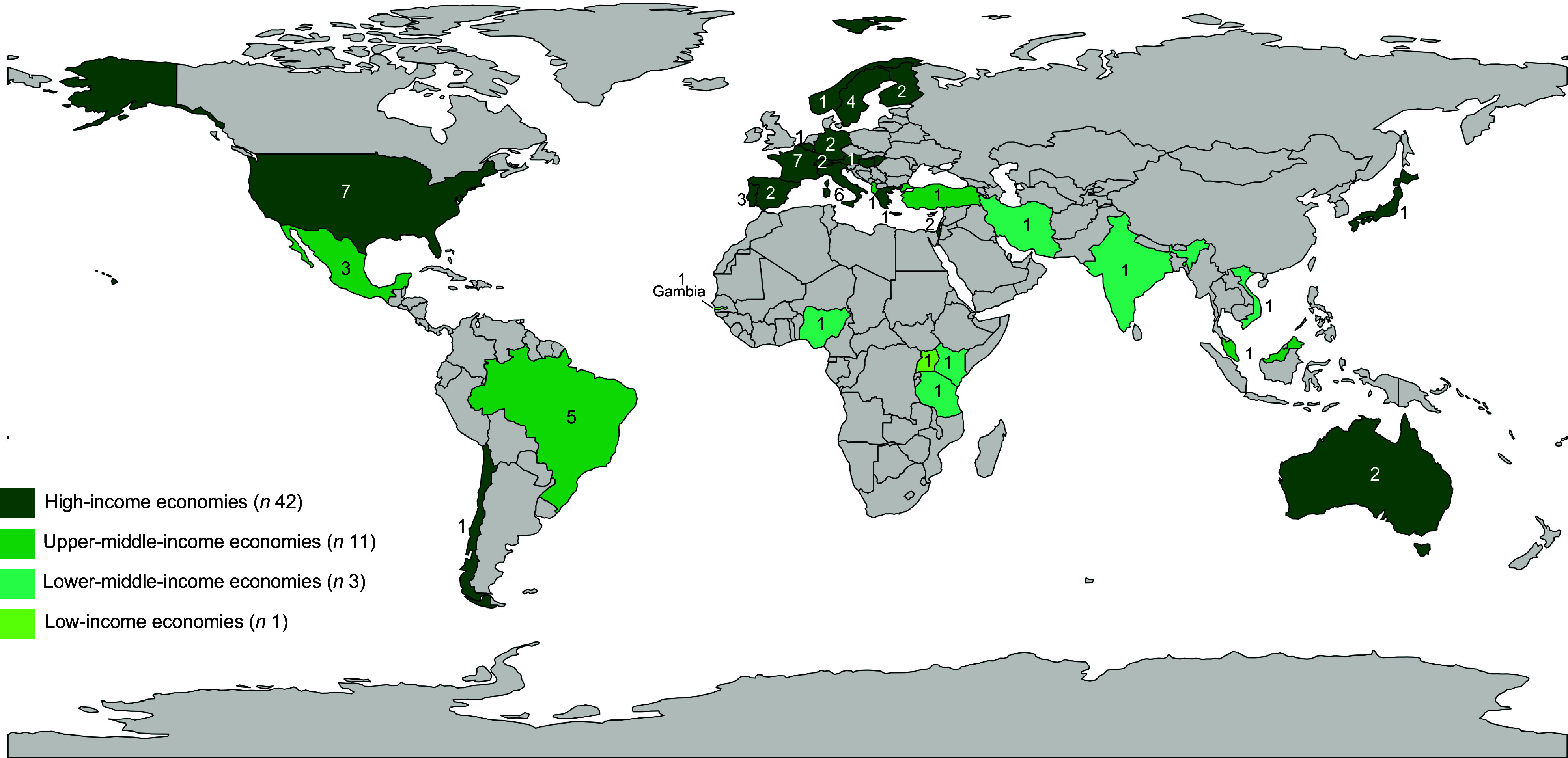
Countries where the studies assessing diet sustainability were carried out Note: The legend shows the number of studies by income level. The map shows studies per country; some studies were conducted in multiple countries.

Among the included studies, 61·4 % utilised and 35·1 % developed an index or tool to assess diet sustainability. As presented in Table 1, regarding the validity of the indices used to assess sustainability, 57·9 % employed validated methods. The majority of studies were published in the last 3 years (71·9 %), employed a cross-sectional design (64·9 %), were conducted in high-income countries (73·7 %) (Figure 2) and involved adults and seniors (77·2 %).

**Table 1.** Characteristics of the studies included in the review (*n* 57)

Assessment of diet sustainability	*n*	%
Uses an index/tool to assess diet sustainability	35	61.40
Develops an index/tool to assess diet sustainability	20	35.09
Develops a sustainable diet concept	2	3·51
**Year of publication**		
2022–2024	41	71·93
2019–2021	14	24·56
2016–2018	2	3·51
**Country**		
High-income economies	42	73·68
Upper-middle-income economies	11	19·30
Lower-middle-income and low-income economies	4	5·26
**Target population**		
Adults	26	45·61
Adults and seniors	18	31·58
Population level	7	12·28
Children and adolescents	4	7·02
Woman only	2	3·51
**Study design**		
Cross-sectional	37	64·91
Cohort	10	17·54
Modeling	5	8·78
Concept development	2	3·51
Observational	2	3·51
Quasi-experimental	1	1·75
**Used a validated index**		
Yes	33	57·90
No	14	24·56
Not applicable (not an index)	10	17·54
**Concept or recommendation of a sustainable diet considered** *		
FAO, 2012	18	31·58
Eat-Lancet Planetary Diet, 2019	37	64·91
Only consider the Eat-Lancet Planetary Diet, 2019	25	43·86
FAO & WHO, 2019	7	12·28
Not specified	4	7·02
Others	1	1·75
**The studies address food processing in any way** †		
No	37	64·91
Yes, directly	11	19·30
Yes, indirectly	9	15·79

* A study could consider more than one sustainable diet concept or recommendation.

† Regarding the approach to food processing, direct assessment refers to studies that explicitly categorised foods by degree of processing or used specific metrics related to the processing stage to identify impacts on diet sustainability; indirect assessment refers to studies that analysed impacts associated with the consumption of industrialised foods, such as discretionary foods, without classifying foods by processing level or using specific metrics related to food processing.

Concerning the concept of a sustainable diet, 43·9 % of the studies explicitly cited an FAO definition (FAO, 2012; FAO & WHO, 2019), while another 43·9 % relied on the recommendations of the EAT-Lancet *Planetary Health Diet* ^(2)^ as a reference, without explicitly framing them in a broader conceptual definition. Overall, 64·9 % of the studies explicitly considered the EAT-Lancet Commission recommendations.

**Figure 3.**
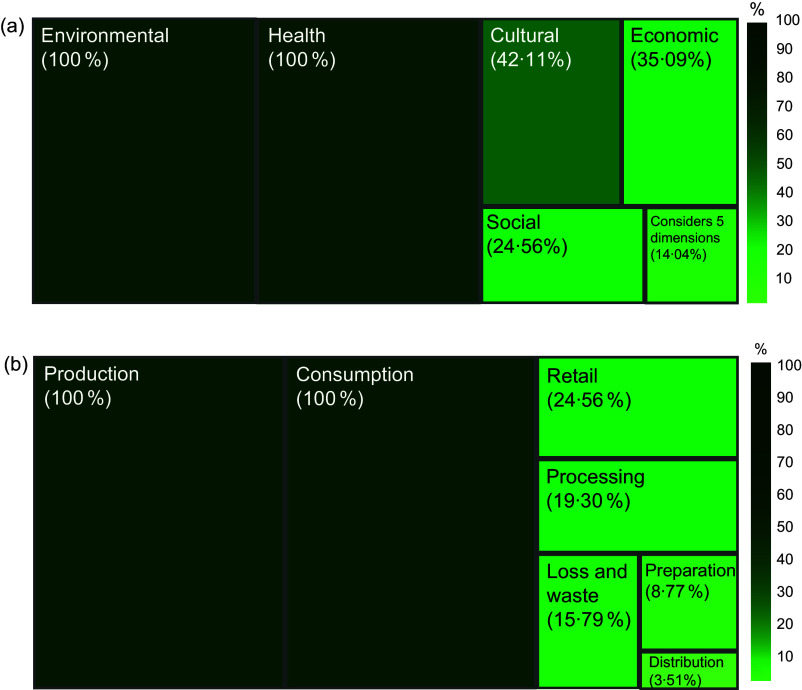
Dimensions (a) and stages (b) of the food system explored in the studies Note: No study explored the impacts of food storage.

Considering the dimensions of the sustainable diet concept, all studies analysed the environmental and health dimensions. However, the cultural (42·1 %) and economic (24·6 %) dimensions were less frequently addressed, while the social dimension was the least explored (24·6 %) (Figure 3a).

Regarding the components of the food systems, production and consumption were the most investigated (100 % of the studies). Trade was examined in 24·6 % of the analyses, while processing was explored in only 19·3 %. Other components received even less attention; food losses and waste were analysed in 15·8 % of the studies, food preparation in 8·8 % and distribution in 3·5 %. No study addressed the impacts of food storage (Figure 3b).

The analysis also revealed a significant gap in the approach to food processing, which was absent in 64·9 % of the studies. Only 19·3 % assessed food processing directly, considering the degree of processing and its associated impacts, or using specific metrics related to the stages of food processing (Table 1). Regarding the classification of foods using the NOVA system, 84·2 % of the studies did not use this approach, and only 8·8 % mentioned the term ‘ultra-processed foods,’ highlighting the absence of this terminology in the analysis.

Among the studies that directly considered food processing, the Sustainable-HEalthy-Diet Index was the most frequently used (*n* 4). This index includes specific components on the consumption of UPF and ready-to-eat meals, focusing primarily on health outcomes and sustainable eating behaviours, while also explicitly acknowledging the social and environmental implications associated with the production and consumption of these products. In addition, one study applied the Healthy and Sustainable Diet Index, which also addressed UPF consumption with a primary emphasis on health impacts, while simultaneously recognising and incorporating environmental consequences (Supplementary Material).

Beyond indices, two conceptual studies addressed food processing directly, emphasising the preference for minimally processed foods as a central element of sustainable dietary patterns. Although one of them reinforces its relevance primarily in relation to dietary quality, Fardet and Rock (2020)^(28)^ proposed a holistic framework in which the degree of processing is treated as a core pillar of sustainability. Finally, other studies that directly evaluated food processing used their own parameters, incorporating UPF consumption into broader analytical frameworks that did not originally include processing (Supplementary Material).

Although an analysis stratified by income level should be interpreted with caution due to the predominance of studies conducted in high-income countries, it appears that studies from low- and lower-middle-income countries did not consider food processing in any way and focused exclusively on the production and consumption stages. In contrast, studies from high-income contexts tended to include additional stages from the food system, particularly trade. Only one study conducted in an upper-middle-income country addressed processing.

**Figure 4.**
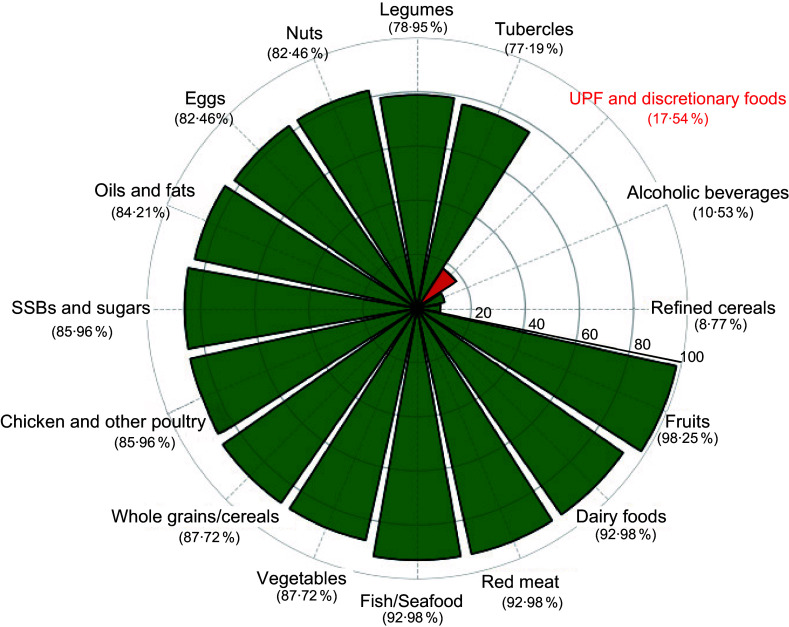
Food groups considered by the studies Note: UPF, ultraprocessed foods; SSB, sugar-sweetened beverages.

In the investigation of food consumption, the most analysed groups were fruits (98·2 %); dairy products, red meat, fish and seafood (all present in 93·0 % of the studies); vegetables (87·7 %) and cereals and whole grains (87·7 %). While 80·7 % investigated the consumption of sugars and sweets, 28·1 % specifically assessed the consumption of sweetened beverages. In contrast, only 17·5 % explored the consumption of ultra-processed or discretionary foods (Figure 4).

Regarding the main results of the studies that developed or applied some index/tool to assess diet sustainability (*n* 55), 30·9 % reported that the diets analysed exhibited only low to moderate levels of sustainability according to the criteria assessed. Furthermore, according to these criteria, 25·4 % found that more sustainable dietary patterns were associated with positive health outcomes (including lower risk of CVD, cancer, obesity and type II diabetes), and 16·4 % identified reductions in environmental impacts according to specific indicators (such as GHG emissions, land use and water use).

Among the main limitations reported by the authors, 38·6 % were related to biases in the methods of consumption assessment (such as self-reporting, memory bias and social desirability bias), 28·1 % referred to sample limitations (including convenience samples, small samples and samples composed mainly of women) and 17·5 % mentioned challenges related to the availability of databases (such as the need for more comprehensive data on foods and environmental impacts tailored to local contexts).

## Discussion

The findings of this review indicate that studies employing indices/tools to assess sustainable diets exhibit a significant gap in addressing intensive food processing, as well as other components of food systems. Moreover, while the environmental and health dimensions are widely explored, the economic and sociocultural dimensions receive considerably less attention. Finally, most studies on this topic have been conducted recently and predominantly in high-income countries.

The analysis of studies aiming to assess sustainable diets reveals a limited view of food systems as a whole, as most of them focus only on the primary production and consumption stages. This limited perspective may underestimate the full range of impacts of UPF across the entire supply chain, since sociocultural and economic effects remain largely unexplored compared to environmental and health dimensions. The scarcity of data, especially considering the isolated use of life cycle assessment as the primary analytical tool, poses a challenge to more comprehensive approaches, as this methodology requires detailed and specific production data, including all stages of the food supply chain and their local characteristics. Such data are often unavailable or restricted to certain foods due to the complexity and multiple intermediaries involved in increasingly globalised food supply chains^(29)^. Furthermore, the lack of standardisation in the use of the term ‘ultra-processed foods’ in studies, as noted in the literature^(8)^, may contribute to an underestimation of the impacts of highly processed foods.

Despite these limitations, particularly the scarcity of comprehensive life-cycle data, the lack of standardised terminology for UPF and the narrow focus of existing analyses, studies emphasise the need to include intensive food processing in the assessment of food sustainability^(8,15,19)^. The literature consistently demonstrates the negative health impacts of highly processed foods and supports the need to limit their consumption^(10,21)^. In addition to health effects, some studies have suggested potential sociocultural and economic implications, such as the loss of culinary traditions and the replacement of local foods, which may negatively affect small-scale producers^(12,14)^. Additionally, economic impacts such as increased healthcare expenditures and premature deaths^(30)^, as well as significant environmental consequences, stemming from reliance on commodities, energy-intensive processing, long transportation chains and heavy use of plastic packaging, have also been documented^(8,9,12,16)^.

Thus, considering the level of food processing, particularly through the NOVA classification, has the potential to provide a more comprehensive understanding of impacts across food systems. By introducing the degree and purpose of industrial processing as an analytical dimension, NOVA can help broaden sustainability assessments by highlighting the processing-related impacts that occur along later stages of the production and supply chain^(31)^. In other words, rather than limiting the analysis to isolated nutrient composition and the impacts of primary production, it is important to consider that, as food becomes more processed, its production tends to require greater use of resources such as energy, water, additives and packaging ^(8,32)^. In this sense, future research could integrate the NOVA classification as a complementary methodological approach to analytical tools such as life cycle assessment, which, although widely used in environmental impact analyses, has limitations in its ability to capture broader aspects of sustainability and dietary patterns^(19)^. Combining NOVA’s sociocultural and health-oriented perspective on food systems with the quantitative environmental estimates provided by such tools could enhance the comprehensiveness and consistency of sustainability assessments.

However, it is important to note that, despite its wide use in the scientific literature and strong epidemiological evidence linking UPF to adverse health outcomes^(10,21)^, the NOVA framework is not without limitations. Challenges remain in its practical application, particularly regarding the classification of heterogeneous foods and the potential for overgeneralisation or inconsistent categorisation^(31)^. Still, as clarified by Monteiro et al. (2018), NOVA aims to identify industrial formulations designed to be hyper-palatable, convenient and profitable, which often come at a high social, environmental and health cost.

Even so, its inclusion in sustainability debates remains relevant, as it expands the analytical scope by incorporating the degree of processing as a complementary dimension alongside other criteria such as nutritional quality, environmental impact, accessibility, economic and cultural aspects. Furthermore, this perspective contributes to a broader assessment of diets by incorporating economic and sociocultural dimensions and avoiding a reductionist bias focused exclusively on nutrient composition^(32)^. The inclusion of the level of processing in the analysis of sustainable diets could also support approaches that, in addition to processing, take a less restrictive view of the food groups assessed, which may not fully reflect the diversity of food systems, especially considering that only 17·5 % of studies include UPF in their group-based assessments.

Despite the potential impacts of UPF across all dimensions of the sustainable diet concept, current studies on sustainability focus predominantly on environmental impacts. In this regard, research has shown that UPF significantly affect environmental metrics such as greenhouse gas emissions, water use and ecological footprint^(33,33–35)^. Moreover, Silva et al. found that the environmental impacts of UPF in Brazil increased over a 30-year period, driven by shifts in dietary patterns towards more processed diets. However, when some studies assess the environmental impact of diets in isolation, and, moreover, when based on impact per unit of energy, the impacts of UPF may be underestimated compared with other food groups, since these products are deliberately formulated to be energy dense and tend to be nutrient poor^(16,36)^. In addition, they rely heavily on resource-intensive commodities and involve intensive industrial processing and packaging use, all of which together underscore the broad negative impacts of these products^(14,37)^. Nonetheless, if environmental impact is considered as the sole parameter, the most effective strategy to reduce dietary environmental burden would be to decrease the consumption of both UPF and animal-based foods simultaneously^(34,38,39)^.

Furthermore, environmental assessments of food systems, which often rely on metrics such as carbon, water and ecological footprints, may overlook critical aspects of agricultural practices. The use of pesticides and chemical fertilisers can cause environmental impacts that extend beyond land occupation, including soil degradation, biodiversity loss, climate change and eutrophication of aquatic ecosystems. These effects are not always captured by conventional footprint-based tools, which tend to focus on land and water use per unit of production^(40,41)^. Therefore, integrating indicators that reflect the broader ecological consequences of food production is essential for a more comprehensive and meaningful sustainability assessment.

In addition to food processing, other components of the production chain, such as storage, food losses and distribution, have been less frequently addressed, especially when compared to production and consumption, which were addressed in all included studies. This gap limits a more comprehensive understanding of the impacts of UPF. It may be attributed not only to the scarcity of data^(19)^ but also to the fact that the most significant impacts are generally associated with primary food production^(3,29)^. This perspective underpinned the recommendations of the EAT-Lancet Commission for the *Planetary Health Diet*, a proposal aimed at promoting health while reducing environmental impact^(2)^, and widely used in studies assessing food sustainability (64·9 %). However, research indicates that post-farm processes can have a substantial impact and should not be overlooked in assessments, regardless of the proportion of UPF in the diet^(8,16)^.

Beyond the limited analysis of food system components, the dimensions encompassed in the FAO’s concept of sustainable diets^(22)^ are also unevenly addressed by assessment tools. While the environmental and health dimensions are frequently explored in analyses, the economic dimension, and especially the sociocultural dimension, is underrepresented, consistent with findings from other reviews^(18,19)^. This gap may compromise the effectiveness of the proposed recommendations, as the acceptance and feasibility of sustainable diets depend on the inclusion of these factors.

One possible explanation for a limited consideration of a comprehensive approach, which considers both all sustainability dimensions and processes beyond primary food production, may be the growing number of studies focused exclusively on assessing adherence to the quantitative recommendations of the *Planetary Health Diet*. In this context, the influence and relevance of the EAT-Lancet Commission’s publication are evident, as it has positively stimulated debate on the topic and provided a framework for developing assessment tools. This is reflected in the predominance of studies that rely solely on the Commission’s recommendations (43·9 %). Furthermore, only a few studies (*n* 5) were identified as incorporating both the FAO’s concept of sustainable diets and the EAT-Lancet recommendations. As research in this area matures, future developments are expected to broaden current approaches, moving beyond the quantitative aspects of dietary composition to more comprehensively incorporate the complex interactions among environmental, economic and sociocultural dimensions of sustainability, including the role of food processing, outlined by both the FAO and the EAT-Lancet Commission^(22,42)^.

This need becomes even more evident in light of the updated EAT–Lancet Commission (2025), which more clearly than its previous version, underscores that the *Planetary Health Diet* should be primarily based on whole, unprocessed and minimally processed foods, ensuring diversity and alignment with local cultures and contexts. In this regard, combining quantitative dietary targets with key qualitative principles of sustainability, as highlighted by the FAO, WHO, and the EAT-Lancet reports^(22,42,43)^, including aspects such as accessibility, desirability and the reduction of food waste, as well as considering a broader systemic perspective, could enhance the comprehensiveness and consistency of future assessments.

These findings are particularly relevant, as evidence suggests that sustainable diets, such as the *Planetary Health Diet*, may be economically inaccessible for certain population groups, especially in low-income countries^(44,45)^, where research remains limited. Moreover, healthy diets based on unprocessed and minimally processed foods, such as fruits and vegetables, may become more expensive in certain regions, making it essential to include access to these foods in the sustainability discourse^(46,47)^, particularly considering the relatively low retail prices of UPF, which are driven by factors such as agricultural subsidies and externalisation of environmental and health costs^(15,17,35)^. On the other hand, some studies show that sustainable diets are less expensive than current dietary patterns in certain settings^(48,49)^. Therefore, taking local particularities into account regarding food access dimensions may be a key strategy for making sustainable diets more affordable and economically viable^(38,50)^. Thus, it is crucial that more studies investigate the main barriers to accessing sustainable diets across diverse populations.

Finally, another contributing factor to the limited exploration of the sociocultural and economic dimensions is the geographical bias observed in other literature reviews on this topic, with a predominant focus on higher-income populations^(19)^, a trend that persists in current studies aiming to assess diet sustainability using any assessment tool^(18)^. In this regard, the results of these studies should not be generalised to all populations, especially considering that this trend limits the understanding of challenges in low- and middle-income contexts, where economic and social barriers play a key role in the accessibility and adherence to sustainable dietary patterns^(22)^, aspects that remain underexplored in the included assessment studies.

Although this scoping review aimed to map the evidence on the role of processing in the assessment and concept of sustainable diets, the literature lacks analyses on the quality of the available evidence. Additionally, despite the comprehensive search strategy employed, the inclusion of studies indexed only in PubMed, Scopus, Web of Science and SciELO, as well as the exclusion of grey literature, may have restricted the breadth of evidence captured and potentially biased the findings towards high-income country contexts by leading to the omission of relevant publications, particularly reports from international organisations, governments, and civil society. Furthermore, the considerable heterogeneity of study designs, populations and assessment approaches limited more in-depth comparisons between studies. Finally, as a scoping review, this study did not aim to evaluate the quality of evidence, which is beyond the scope of this methodological design. Nevertheless, the study contributes to reflections on the need for a more comprehensive, inclusive and contextually adapted approach to the assessment of sustainable diets across diverse populations.

This review emphasises the need for a more comprehensive understanding of the assessment of sustainable diets, particularly regarding the impacts of UPF. The development of tools and methodologies that consider the intensive processing of foods in sustainability assessments is necessary, aiming for more comprehensive practical analyses that encompass the entire food chain and address the challenge of incorporating economic and sociocultural aspects and their interrelations, beyond health and environmental indicators. It is essential to invest in the generation of local data, enabling a more precise understanding of the specific impacts of diverse food systems and, most importantly, of the barriers and opportunities for the adoption of sustainable diets in different contexts.

Future research should move beyond descriptive approaches and focus on operationalising these advances. This includes integrating the NOVA classification into existing indices and life cycle assessment frameworks to capture processing-related impacts more effectively, as well as systematically incorporating local economic constraints and cultural dimensions, such as food traditions and preferences, into sustainability assessments. Moreover, as most evidence comes from high-income countries, expanding research in low- and middle-income countries, especially studies comparing income and access to food, is essential to improve the representativeness and applicability of sustainable diet assessments across diverse contexts. Advancing research in these directions would enable the development of more context-sensitive tools capable of informing comprehensive policies and guiding practical interventions to promote healthier and more sustainable food systems.

## Conclusion

This review reinforces the need for a more comprehensive approach to evaluating sustainable diets, one that incorporates not only environmental and health impacts, but also economic and sociocultural dimensions. The level of food processing should be systematically considered, as it influences the entire production chain and affects multiple aspects of the food system sustainability. Furthermore, it is essential to broaden the scope of sustainability research to assess other components of food systems, such as storage, distribution, food losses and waste and trade, which remain underexplored.

The development of tools and studies that embrace this integrated approach can support more equitable public policies, tailored to regional specificities and promote food systems that respect cultural traditions, support local producers and ensure equitable access to healthy foods.

## Supporting information

Cordeiro et al. supplementary material Cordeiro et al. supplementary material

## Supplementary material

To view supplementary material for this article, please visit https://doi.org/10.1017/S1368980026102237
