# Supplementary material for: Do sustainable diets take food processing into account? A scoping review
Source: Public Health Nutr. 2026 Mar 3;29(1):e73. doi: 10.1017/S1368980026102237 (PMC13087983; doi:10.1017/S1368980026102237)
Supplement: Cordeiro et al. supplementary material [file S1368980026102237sup001.docx]

**SUPPLEMENTARY MATERIAL**

**1. Search strategy and keywords**

| **Topic** | **Terms searched in the databases** |
| --- | --- |
| **Sustainable diet** | "Sustainable Diets" OR “Sustainable Diet" OR “Healthy and Sustainable Diet” OR "Dietary Sustainability" OR "Food Sustainability" OR "Sustainable Nutrition" OR "Sustainable Eating" OR "Environmentally Sustainable Diets" |
| **Indices/tools for assessing the sustainability of diets** | “Sustainable Diet Index” OR “Healthy and Sustainable Diet Index” OR Food Sustainability Index” OR “Dietary Sustainability Metrics” OR “Sustainable Diet Assessment” OR “Diet Sustainability Indicators” |
| **Concept of sustainable diets** | “Sustainable Diet Concept” OR “Food Sustainability Concept” OR “Healthy and Sustainable Diet Concept” OR “Sustainable Diet Model” OR “Food Sustainability Model” OR “Healthy and Sustainable Diet Model” OR “Sustainable Diet Principle” OR “Food Sustainability Principle” OR “Healthy and Sustainable Diet Principle” OR “Sustainable Diet Paradigm” OR “Food Sustainability Paradigm” OR “Healthy and Sustainable Diet Paradigm” OR “Sustainable Diet Theory” OR “Food Sustainability Theory” OR “Healthy and Sustainable Diet Theory” |

**2. Description of the articles included in the scoping review and the main data extracted from each one (n=57).**

| **First author**  **(year of publication)** | **Journal name** | **Country** | **Assessment of diet sustainability** | **Target population** | **Study design** | **Concept or recommendation of a sustainable diet considered** | **Address food processing in any way** | **Used a validated index** |
| --- | --- | --- | --- | --- | --- | --- | --- | --- |
| Keding (2023)^1^ | Nutrients | Kenya, Tanzania & Uganda | Uses an index/tool | Woman | Cross-sectional | Not specified | No | Yes |
| Bjørnarå (2019)^2^ | Scand J Public Health. | Norway | Develops an index/tool | Population level | Cross-sectional | FAO, 2012 | No | No |
| Berthy  (2023)^3^ | Am J Clin Nutr | France | Uses an index/tool | Adults | Cross-sectional | Eat-Lancet | Yes, directly | No |
| Fardet  (2020)^4^ | Public Health Nutr | France | Develops a concept | Population level | Concept development | N/A | Yes, directly | N/A |
| Marchioni  (2022)^5^ | Nutrients | Brazil | Uses an index/tool | Population level | Cross-sectional | Eat-Lancet | No | Yes |
| Trijsburg  (2020)^6^ | Nutrients | Vietnam & Nigeria | Uses an index/tool | Adults | Cross-sectional | Eat-Lancet | No | Yes |
| Ruggeri  (2022)^7^ | Nutrients | Italy | Develops a concept | Adults and elderly | Cross-sectional | Eat-Lancet | No | Yes |
| Ludwig  (2023)^8^ | Public Health Nutr | USA | Uses an index/tool | Adults | Cross-sectional | Eat-Lancet | No | No |
| Seconda  (2020)^9^ | Eur J Epidemiol | France | Uses an index/tool | Adults and elderly | Prospective cohort | FAO, 2012 | Yes, indirectly | Yes |
| Cacau  (2023)^10^ | Eur J Nutr | Greece, Germany, Belgium, France, Hungary, Italy, Sweden, Austria & Spain | Uses an index/tool | Adolescents | Cross-sectional | FAO & WHO, 2019 | No | Yes |
| Navruz  (2023)^11^ | Nutrients | Turkey | Uses an index/tool | Adults | Observational | Eat-Lancet | No | Yes |
| Bäck  (2022)^12^ | Eur J Nutr | Finland | Uses an index/tool | Children | Cross-sectional | Eat-Lancet | No | N/A |
| Curi-Quinto  (2022)^13^ | Front Nutr | Mexico | Uses an index/tool | Adults | Cross-sectional | FAO, 2012 | No | N/A |
| Jung  (2022)^14^ | Int J Epidemiol | USA | Uses an index/tool | Adults | Observational prospective | FAO, 2012 | Yes, indirectly | Yes |
| Campirano  (2022)^15^ | Nutrients | Mexico | Develops an index/tool | Adults | Cross-sectional | Eat-Lancet | No | No |
| Zhang  (2024)^16^ | ACC Heart Fail | Sweden | Uses an index/tool | Adults and elderly | Prospective cohort | Eat-Lancet | No | Yes |
| Zhang  (2023) ^17^ | BMC Med | Sweden | Uses an index/tool | Adults and elderly | Prospective cohort | Eat-Lancet | No | Yes |
| Tepper  (2022)^18^ | Front Nutr | Israel | Uses an index/tool | Adults | Cross-sectional | Not specified | Yes, directly | No |
| Rodrigues  (2024)^19^ | Nutrients | Portugal | Uses an index/tool | Children | Cross-sectional | FAO & WHO, 2019; Eat-Lancet | No | No |
| Jafari  (2024)^20^ | Br J Nutr | Iran | Uses an index/tool | Woman | Cross-sectional | FAO, 2012; Eat-Lancet | No | Yes |
| Zanatta  (2022)^21^ | Foods | Italy | Uses an index/tool | Adults and elderly | Cross-sectional | FAO & WHO, 2019; Eat-Lancet | No | No |
| Tepper  (2021)^22^ | Eur J Nutr | Israel | Develops an index/tool | Adults | Cross-sectional | FAO, 2012 | Yes, directly | Yes |
| Martins (2023)^23^ | Nutrients | Portugal | Uses an index/tool | Adults | Cross-sectional | FAO & WHO, 2019 | Yes, directly | Yes |
| Donati  (2016)^24^ | Appetite | Italy | Uses an index/tool | Adults | Mathematical modeling | FAO, 2012 | No | N/A |
| Hendrie  (2022)^25^ | BMC Public Health | Australia | Uses an index/tool | Adults | Cross-sectional | FAO & WHO, 2019; Eat-Lancet | Yes, indirectly | N/A |
| Agyemang  (2022)^26^ | Front Nutr | USA | Develops an index/tool | Population level | Mathematical modeling | FAO, 2012; Eat-Lancet | No | N/A |
| Minotti  (2022)^27^ | Front Nutr | Italy | Uses an index/tool | Population level | Mathematical modeling | Not specified | No | N/A |
| Zulkefli  (2022)^28^ | Malaysian Journal of Nutrition | Malaysia | Develops an index/tool | Adults | Cross-sectional | FAO, 2012 | No | Yes |
| Koerber  (2017)^29^ | Proc Nutr Soc | Germany | Develops a concept | Population level | Concept development | N/A | Yes, directly | N/A |
| Tucci  (2024)^30^ | Curr Res Food Sci | Italy | Develops an index/tool | Adults | Mathematical modeling | FAO, 2012; FAO & WHO, 2019; Eat-Lancet | Yes, directly | N/A |
| Fresán  (2020)^31^ | Prev Med | Spain | Develops an index/tool | Adults | Prospective cohort | FAO, 2012 | Yes, directly | Yes |
| Venegas  (2023)^32^ | Int J Behav Nutr | Chile | Develops an index/tool | Children | Cross-sectional | FAO & WHO, 2019; Eat-Lancet | No | No |
| Ali  (2022)^33^ | Environ Res Lett | Gambia | Develops an index/tool | Population level | Cross-sectional | Eat-Lancet | No | No |
| Llanaj  (2021)^34^ | Br J Nutr | Albania | Uses an index/tool | Adults | Cross-sectional | Eat-Lancet | No | No |
| Zhang  (2023)^35^ | Am J Clin Nutr | Sweden | Uses an index/tool | Adults and elderly | Prospective cohort | Eat-Lancet | No | Yes |
| Pitt  (2024)^36^ | Environ Int | Sweden | Develops an index/tool | Adults and elderly | Prospective cohort | Eat-Lancet | No | Yes |
| Zhang  (2023)^37^ | Metabolism | Sweden | Uses an index/tool | Adults and elderly | Prospective cohort | Eat-Lancet | No | Yes |
| Cacau  (2023)^38^ | Eur J Nutr | Brazil | Uses an index/tool | Adults and elderly | Cross-sectional | Eat-Lancet | No | Yes |
| Franchini  (2024)^39^ | Nutr J | USA | Uses an index/tool | Adults | Cross-sectional | Eat-Lancet | Yes, directly | Yes |
| Frank  (2024)^40^ | Am J Clin Nutr | USA | Uses an index/tool | Adults | Cross-sectional | Eat-Lancet | No | Yes |
| Cacau  (2021)^41^ | Nutrients | Brazil | Uses an index/tool | Adults and elderly | Cross-sectional | Eat-Lancet | No | Yes |
| Berthy  (2022)^42^ | Am J Clin Nutr | France | Uses an index/tool | Adults and elderly | Prospective cohort | Eat-Lancet | Yes, indirectly | Yes |
| Seconda  (2020)^43^ | Am J Clin Nutr | France | Uses an index/tool | Adults and elderly | Prospective cohort | Eat-Lancet | Yes, indirectly | Yes |
| Shamah-Levy  (2020)^44^ | Salud Publica Mex | Mexico | Develops an index/tool | Adults | Cross-sectional | Eat-Lancet | No | No |
| Baudry  (2022)^45^ | Public Health Nutr | France | Uses an index/tool | Adults and elderly | Cross-sectional | FAO, 2012 | Yes, indirectly | Yes |
| Suikki  (2023)^46^ | Food Nutr Res | Finland | Develops an index/tool | Adults | Cohort | Eat-Lancet | No | No |
| Jung  (2024)^47^ | Nutr J | USA | Develops an index/tool | Adults | Cross-sectional | FAO, 2012; Eat-Lancet | Yes, indirectly | Yes |
| Chen  (2019)^48^ | Nutrients | Switzerland | Uses an index/tool | Adults | Cross-sectional | FAO, 2012 | No | Yes |
| Harrison  (2024)^49^ | Nutr J | Germany | Uses an index/tool | Adults and elderly | Quasi-experimental | Eat-Lancet | No | No |
| Ganpule  (2023)^50^ | J Hum Nutr Diet | India | Develops an index/tool | Adults | Cross-sectional | Eat-Lancet | No | Yes |
| Bôto  (2024)^51^ | Sustainable Production and Consumption | Portugal | Develops an index/tool | Adults | Cross-sectional | FAO, 2012 | Yes, indirectly | Yes |
| Cacau  (2021)^52^ | Nutrients | Brazil | Develops an index/tool | Adults and elderly | Cross-sectional | Eat-Lancet | No | Yes |
| Frank  (2021)^53^ | Int J Behav Nutr Phys Act | USA | Uses an index/tool | Adults | Cross-sectional | Eat-Lancet | No | Yes |
| Seconda  (2019)^54^ | Br J Nutr | France | Develops an index/tool | Adults and elderly | Cross-sectional | FAO, 2012 | Yes, indirectly | Yes |
| Sugimoto  (2022)^55^ | Br J Nutr | Japan | Uses an index/tool | Adults and elderly | Cross-sectional | FAO, 2012; Eat-Lancet; Tilman & Clark, 2014 | No | No |
| Harray  (2022)^56^ | Nutrients | Australia | Develops an index/tool | Adults and elderly | Cross-sectional | FAO, 2012; Eat-Lancet; | Yes, directly | Yes |
| Caldeira  (2024)^57^ | Nutr J | Brazil | Uses an index/tool | Adults | Mathematical modeling | Not specified | Yes, directly | N/A |

1. Keding GB, Sarfo J, Pawelzik E. Healthy Diets from Sustainable Food Systems: Calculating the WISH Scores for Women in Rural East Africa. Nutrients. 2023;15(12):2699. Published 2023 Jun 9. doi:10.3390/nu15122699

2. Bjørnarå HB, Torstveit MK, Bere E. Healthy and sustainable diet and physical activity: the rationale for and experiences from developing a combined summary score. Scand J Public Health. 2019;47(5):583-591. doi:10.1177/1403494818785056

3. Berthy F, Brunin J, Allès B, et al. Higher adherence to the EAT-Lancet reference diet is associated with higher nutrient adequacy in the NutriNet-Santé cohort: a cross-sectional study. Am J Clin Nutr. 2023;117(6):1174-1185. doi:10.1016/j.ajcnut.2023.03.029

4. Fardet A, Rock E. How to protect both health and food system sustainability? A holistic 'global health'-based approach via the 3V rule proposal. Public Health Nutr. 2020;23(16):3028-3044. doi:10.1017/S136898002000227X

5. Marchioni DM, Cacau LT, De Carli E, Carvalho AM, Rulli MC. Low Adherence to the EAT-Lancet Sustainable Reference Diet in the Brazilian Population: Findings from the National Dietary Survey 2017-2018. Nutrients. 2022;14(6):1187. Published 2022 Mar 11. doi:10.3390/nu14061187

6. Trijsburg L, Talsma EF, Crispim SP, et al. Method for the Development of WISH, a Globally Applicable Index for Healthy Diets from Sustainable Food Systems. Nutrients. 2020;13(1):93. Published 2020 Dec 30. doi:10.3390/nu13010093

7. Ruggeri S, Buonocore P, Amoriello T. New Validated Short Questionnaire for the Evaluation of the Adherence of Mediterranean Diet and Nutritional Sustainability in All Adult Population Groups. Nutrients. 2022;14(23):5177. Published 2022 Dec 5. doi:10.3390/nu14235177

8. Ludwig-Borycz E, Neumark-Sztainer D, Larson N, et al. Personal, behavioural and socio-environmental correlates of emerging adults' sustainable food consumption in a cross-sectional analysis. Public Health Nutr. 2023;26(6):1306-1316. doi:10.1017/S1368980023000654

9. Seconda L, Baudry J, Allès B, et al. Prospective associations between sustainable dietary pattern assessed with the Sustainable Diet Index (SDI) and risk of cancer and cardiovascular diseases in the French NutriNet-Santé cohort. Eur J Epidemiol. 2020;35(5):471-481. doi:10.1007/s10654-020-00619-2

10. Cacau LT, Hanley-Cook GT, Huybrechts I, et al. Relative validity of the Planetary Health Diet Index by comparison with usual nutrient intakes, plasma food consumption biomarkers, and adherence to the Mediterranean diet among European adolescents: the HELENA study. Eur J Nutr. 2023;62(6):2527-2539. doi:10.1007/s00394-023-03171-3

11. Navruz-Varlı S, Mortaş H. Shift Work, Shifted Diets: An Observational Follow-Up Study on Diet Quality and Sustainability among Healthcare Workers on Night Shifts. Nutrients. 2024;16(15):2404. Published 2024 Jul 24. doi:10.3390/nu16152404

12. Bäck S, Skaffari E, Vepsäläinen H, et al. Sustainability analysis of Finnish pre-schoolers' diet based on targets of the EAT-Lancet reference diet. Eur J Nutr. 2022;61(2):717-728. doi:10.1007/s00394-021-02672-3

13. Curi-Quinto K, Unar-Munguía M, Rodríguez-Ramírez S, et al. Sustainability of Diets in Mexico: Diet Quality, Environmental Footprint, Diet Cost, and Sociodemographic Factors. Front Nutr. 2022;9:855793. Published 2022 May 27. doi:10.3389/fnut.2022.855793

14. Jung S, Young HA, Simmens SJ, Braffett BH, Ogden CL. Sustainable dietary patterns and all-cause mortality among US adults. Int J Epidemiol. 2024;53(1):dyad176. doi:10.1093/ije/dyad176

15. Campirano F, López-Olmedo N, Ramírez-Palacios P, Salmerón J. Sustainable Dietary Score: Methodology for Its Assessment in Mexico Based on EAT-Lancet Recommendations. Nutrients. 2023;15(4):1017. Published 2023 Feb 17. doi:10.3390/nu15041017

16. Zhang S, Marken I, Stubbendorff A, et al. The EAT-Lancet Diet Index, Plasma Proteins, and Risk of Heart Failure in a Population-Based Cohort. JACC Heart Fail. 2024;12(7):1197-1208. doi:10.1016/j.jchf.2024.02.017

17. Zhang S, Stubbendorff A, Ericson U, et al. The EAT-Lancet diet, genetic susceptibility and risk of atrial fibrillation in a population-based cohort. BMC Med. 2023;21(1):280. Published 2023 Jul 28. doi:10.1186/s12916-023-02985-6

18. Tepper S, Kissinger M, Avital K, Shahar DR. The Environmental Footprint Associated With the Mediterranean Diet, EAT-Lancet Diet, and the Sustainable Healthy Diet Index: A Population-Based Study. Front Nutr. 2022;9:870883. Published 2022 May 19. doi:10.3389/fnut.2022.870883

19. Rodrigues M, Padrão P, Castro Mendes F, Moreira A, Moreira P. The Planetary Health Diet and Its Association with Asthma and Airway Inflammation in School-Aged Children. Nutrients. 2024;16(14):2241. Published 2024 Jul 12. doi:10.3390/nu16142241

20. Jafari A, Lotfi K, Mozaffari H, et al. The relationship between the World Index for Sustainability and Health (WISH) score and mental health in women: a cross-sectional study. Br J Nutr. 2024;132(2):151-161. doi:10.1017/S0007114524000771

21. Zanatta F, Mari S, Adorni R, et al. The Role of Selected Psychological Factors in Healthy-Sustainable Food Consumption Behaviors during the COVID-19 Pandemic. Foods. 2022;11(13):1944. Published 2022 Jun 29. doi:10.3390/foods11131944

22. Tepper S, Geva D, Shahar DR, et al. The SHED Index: a tool for assessing a Sustainable HEalthy Diet. Eur J Nutr. 2021;60(7):3897-3909. doi:10.1007/s00394-021-02554-8

23. Liz Martins M, Tepper S, Marques B, Abreu S. The SHED Index: A Validation Study to Assess Sustainable HEalthy Diets in Portugal. Nutrients. 2023;15(24):5071. Published 2023 Dec 12. doi:10.3390/nu15245071

24. Donati M, Menozzi D, Zighetti C, Rosi A, Zinetti A, Scazzina F. Towards a sustainable diet combining economic, environmental and nutritional objectives. Appetite. 2016;106:48-57. doi:10.1016/j.appet.2016.02.151

25. Hendrie GA, Rebuli MA, James-Martin G, et al. Towards healthier and more sustainable diets in the Australian context: comparison of current diets with the Australian Dietary Guidelines and the EAT-Lancet Planetary Health Diet. BMC Public Health. 2022;22(1):1939. Published 2022 Oct 19. doi:10.1186/s12889-022-14252-z

26. Agyemang P, Kwofie EM, Baum JI. Transitioning to sustainable healthy diets: A model-based and conceptual system thinking approach to optimized sustainable diet concepts in the United States. Front Nutr. 2022;9:874721. Published 2022 Jul 29. doi:10.3389/fnut.2022.874721

27. Minotti B, Antonelli M, Dembska K, et al. True Cost Accounting of a healthy and sustainable diet in Italy. Front Nutr. 2022;9:974768. Published 2022 Jul 29. doi:10.3389/fnut.2022.974768

28. Zulkefli, NF, & Moy, FM. Validation of a Sustainable Diet Index among young Malaysian adults. Malaysian Journal of Nutrition. 2022;28(2). doi.org/10.31246/mjn-2021-0060

29. Koerber VK, Bader N, Leitzmann C. Wholesome Nutrition: an example for a sustainable diet. Proc Nutr Soc. 2017;76(1):34-41. doi:10.1017/S0029665116000616

30. Tucci M, Martini D, Vinelli V, et al. The MED_EAT-IT approach: A modelling study to develop feasible, sustainable and nutritionally targeted dietary patterns based on the Planetary health diet. Curr Res Food Sci. 2024;8:100765. Published 2024 May 7. doi:10.1016/j.crfs.2024.100765

31. Fresán U, Martínez-González MA, Segovia-Siapco G, Sabaté J, Bes-Rastrollo M. A three-dimensional dietary index (nutritional quality, environment and price) and reduced mortality: The "Seguimiento Universidad de Navarra" cohort. Prev Med. 2020;137:106124. doi:10.1016/j.ypmed.2020.106124

32. Venegas Hargous C, Orellana L, Strugnell C, Corvalan C, Allender S, Bell C. Adapting the Planetary Health Diet Index for children and adolescents. Int J Behav Nutr Phys Act. 2023;20(1):146. Published 2023 Dec 14. doi:10.1186/s12966-023-01516-z

33. Ali Z, Scheelbeek PFD, Felix J, et al. Adherence to EAT-Lancet dietary recommendations for health and sustainability in the Gambia. Environ Res Lett. 2022;17(10):104043. doi:10.1088/1748-9326/ac9326

34. Llanaj E, Hanley-Cook GT. Adherence to healthy and sustainable diets is not differentiated by cost, but rather source of foods among young adults in Albania. Br J Nutr. 2021;126(4):591-599. doi:10.1017/S0007114520004390

35. Zhang S, Dukuzimana J, Stubbendorff A, Ericson U, Borné Y, Sonestedt E. Adherence to the EAT-Lancet diet and risk of coronary events in the Malmö Diet and Cancer cohort study. Am J Clin Nutr. 2023;117(5):903-909. doi:10.1016/j.ajcnut.2023.02.018

36. Pitt S, Kałuża J, Widenfalk A, Åkesson A, Wolk A. Adherence to the EAT-Lancet diet in relation to mortality and exposure to food contaminants in population-based cohorts of Swedish men and women. Environ Int. 2024;184:108495. doi:10.1016/j.envint.2024.108495

37. Zhang S, Stubbendorff A, Olsson K, et al. Adherence to the EAT-Lancet diet, genetic susceptibility, and risk of type 2 diabetes in Swedish adults. Metabolism. 2023;141:155401. doi:10.1016/j.metabol.2023.155401

38. Cacau LT, Benseñor IM, Goulart AC, et al. Adherence to the EAT-Lancet sustainable reference diet and cardiometabolic risk profile: cross-sectional results from the ELSA-Brasil cohort study. Eur J Nutr. 2023;62(2):807-817. doi:10.1007/s00394-022-03032-5

39. Franchini C, Biasini B, Sogari G, et al. Adherence to the Mediterranean Diet and its association with sustainable dietary behaviors, sociodemographic factors, and lifestyle: a cross-sectional study in US University students. Nutr J. 2024;23(1):56. Published 2024 May 27. doi:10.1186/s12937-024-00962-0

40. Frank SM, Jaacks LM, Adair LS, et al. Adherence to the Planetary Health Diet Index and correlation with nutrients of public health concern: an analysis of NHANES 2003-2018. Am J Clin Nutr. 2024;119(2):384-392. doi:10.1016/j.ajcnut.2023.10.018

41. Cacau LT, Benseñor IM, Goulart AC, et al. Adherence to the Planetary Health Diet Index and Obesity Indicators in the Brazilian Longitudinal Study of Adult Health (ELSA-Brasil). Nutrients. 2021;13(11):3691. Published 2021 Oct 20. doi:10.3390/nu13113691

42. Berthy F, Brunin J, Allès B, et al. Association between adherence to the EAT-Lancet diet and risk of cancer and cardiovascular outcomes in the prospective NutriNet-Santé cohort. Am J Clin Nutr. 2022;116(4):980-991. doi:10.1093/ajcn/nqac208

43. Seconda L, Egnell M, Julia C, et al. Association between sustainable dietary patterns and body weight, overweight, and obesity risk in the NutriNet-Santé prospective cohort. Am J Clin Nutr. 2020;112(1):138-149. doi:10.1093/ajcn/nqz259

44. Shamah-Levy T, Gaona-Pineda EB, Mundo-Rosas V, Méndez Gómez-Humarán I, Rodríguez-Ramírez S. Asociación de un índice de dieta saludable y sostenible con sobrepeso y obesidad en adultos mexicanos. Salud Publica Mex. 2020;62(6):745-753. doi:10.21149/11829

45. Baudry J, Allès B, Langevin B, et al. Associations between measures of socio-economic position and sustainable dietary patterns in the NutriNet-Santé study. Public Health Nutr. 2022;26(5):965-975. doi:10.1017/S1368980022002208

46. Suikki T, Maukonen M, Kaartinen NE, et al. Associations of EAT-Lancet Planetary Health Diet or Finnish Nutrition Recommendations with changes in obesity measures: a follow-up study in adults. Food Nutr Res. 2023;67. Published 2023 Dec 1. doi:10.29219/fnr.v67.9107

47. Jung S, Young HA, Braffett BH, Simmens SJ, Ogden CL. Development of a sustainable diet index in US adults. Nutr J. 2024;23(1):46. Published 2024 Apr 10. doi:10.1186/s12937-024-00943-3

48. Chen C, Chaudhary A, Mathys A. Dietary Change Scenarios and Implications for Environmental, Nutrition, Human Health and Economic Dimensions of Food Sustainability. Nutrients. 2019;11(4):856. Published 2019 Apr 16. doi:10.3390/nu11040856

49. Harrison L, Herrmann A, Quitmann C, et al. Effects of a cafeteria-based sustainable diet intervention on the adherence to the EAT-Lancet planetary health diet and greenhouse gas emissions of consumers: a quasi-experimental study at a large German hospital. Nutr J. 2024;23(1):80. Published 2024 Jul 18. doi:10.1186/s12937-024-00981-x.

50. Ganpule A, Dubey M, Pandey H, et al. Dietary patterns in North and South India: a comparison with EAT-Lancet dietary recommendations. J Hum Nutr Diet. 2023;36(6):2170-2179. doi:10.1111/jhn.13222

51. Bôto, J. M., Neto, B., Miguéis, V., & Rocha, A.Development of the Dietary Pattern Sustainability Index (DIPASI): A novel multidimensional approach for assessing the sustainability of an individual's diet. Sustainable Production and Consumption. 2024;50, 139-154. doi.org/10.1016/j.spc.2024.07.029

52. Cacau LT, De Carli E, de Carvalho AM, et al. Development and Validation of an Index Based on EAT-Lancet Recommendations: The Planetary Health Diet Index. Nutrients. 2021;13(5):1698. Published 2021 May 17. doi:10.3390/nu13051698

53. Frank SM, Jaacks LM, Meyer K, et al. Dietary quality and dietary greenhouse gas emissions in the USA: a comparison of the planetary health diet index, healthy eating index-2015, and dietary approaches to stop hypertension. Int J Behav Nutr Phys Act. 2024;21(1):36. Published 2024 Apr 2. doi:10.1186/s12966-024-01581-y

54. Seconda L, Baudry J, Pointereau P, et al. Development and validation of an individual sustainable diet index in the NutriNet-Santé study cohort. Br J Nutr. 2019;121(10):1166-1177. doi:10.1017/S0007114519000369

55. Sugimoto M, Temme EHM, Biesbroek S, et al. Exploring culturally acceptable, nutritious, affordable and low climatic impact diet for Japanese diets: proof of concept of applying a new modelling approach using data envelopment analysis. Br J Nutr. 2022;128(12):2438-2452. doi:10.1017/S0007114522000095

56. Harray AJ, Boushey CJ, Pollard CM, et al. Healthy and Sustainable Diet Index: Development, Application and Evaluation Using Image-Based Food Records. Nutrients. 2022;14(18):3838. Published 2022 Sep 16. doi:10.3390/nu14183838

57. Caldeira TCM, Vandevijvere S, Swinburn B, Mackay S, Claro RM. Differences in the cost and environmental impact between the current diet in Brazil and healthy and sustainable diets: a modeling study. Nutr J. 2024;23(1):71. Published 2024 Jul 9. doi:10.1186/s12937-024-00973-x
